# Diffusion tensor imaging correlates with cytopathology in a rat model of neonatal hydrocephalus

**DOI:** 10.1186/1743-8454-7-19

**Published:** 2010-11-05

**Authors:** Weihong Yuan, Kelley E Deren, James P McAllister, Scott K Holland, Diana M Lindquist, Alessandro Cancelliere, Melissa Mason, Ahmed Shereen, Dean A Hertzler, Mekibib Altaye, Francesco T Mangano

**Affiliations:** 1 Department of Radiology, Pediatric Neuroimaging Research Consortium, Cincinnati Children's Hospital Medical Center, University of Cincinnati College of Medicine, MLC 5033, 3333 Burnet Ave., Cincinnati, OH 45229, USA; 2 Department of Neurosurgery, Division of Pediatric Neurosurgery, Primary Children's Medical Center and the University of Utah, N. Medical Drive East, Salt Lake City, UT 84132, USA; 3 Department of Radiology, Imaging Research Center, Cincinnati Children's Hospital Medical Center and University of Cincinnati College of Medicine, MLC 5033, 3333 Burnet Ave., Cincinnati, OH 45229, USA; 4 Division of Pediatric Neurosurgery, University of Cincinnati, Cincinnati Children's Hospital Medical Center MLC 2016, 3333 Burnet Avenue, Cincinnati, OH 45229, USA; 5 Department of Biostatistics & Epidemiology, Cincinnati Children's Hospital Medical Center, MLC 5041, 3333 Burnet Ave., Cincinnati, OH 45229, USA

## Abstract

**Background:**

Diffusion tensor imaging (DTI) is a non-invasive MRI technique that has been used to quantify CNS abnormalities in various pathologic conditions. This study was designed to quantify the anisotropic diffusion properties in the brain of neonatal rats with hydrocephalus (HCP) and to investigate association between DTI measurements and cytopathology.

**Methods:**

DTI data were acquired between postnatal day 7 (P7) and P12 in 12 rats with HCP induced at P2 and in 15 age-matched controls. Animals were euthanized at P11 or P22/P23 and brains were processed with immunohistochemistry for glial fibrillary acidic protein (GFAP), ionized calcium-binding adaptor molecule (Iba-1), and luxol fast blue (LFB) to assess astrocytosis, microglial reactivity and degree of myelination, respectively.

**Results:**

Hydrocephalic rats were consistently found to have an abnormally low (at corrected *p*-level of <0.05) fractional anisotropy (FA) value and an abnormally high mean diffusivity (MD) value in the cerebral cortex (CX), the corpus callosum (CC), and the internal capsule (IC). Immunohistochemical analysis demonstrated trends of increasing astrocyte and microglial reactivity in HCP rats at P11 that reached statistical significance at P22/P23. A trend toward reduced myelination in the HCP rats was also found at P22/P23. Correlation analysis at P11 for the CC demonstrated statistically significant correlations (or trends) between the DTI measurement (the decreased FA and increased MD values) and the GFAP or Iba-1 rankings. The immunohistochemical rankings in the IC at P22/P23 were also significantly correlated or demonstrated a trend with both FA and MD values.

**Conclusions:**

This study demonstrates the feasibility of employing DTI on the brain in experimental hydrocephalus in neonatal rats and reveals impairments in multiple regions of interest in both grey and white matter. A strong correlation was found between the immunohistochemical results and the changes in anisotropic diffusion properties.

## Background

Hydrocephalus (HCP) is the most common condition treated in pediatric neurosurgery [1]. The ventricular system enlarges in response to abnormal cerebrospinal fluid (CSF) dynamics. As a consequence of this imbalance, intra-cranial pressure becomes elevated, ventricles enlarge, and brain anatomy becomes distorted and compressed. If left untreated HCP may be fatal [1]. However, long before this end-point is reached, injury to grey matter (GM) and white matter (WM) structures can cause significant neurologic deficits. Behavioral and cognitive delays typically accompany the brain abnormalities associated with HCP. The current standard of care is based on CSF diversion to extra-neural compartments via shunt surgery or via third ventriculostomy. Even though this approach has greatly reduced morbidity and mortality, much of the pathophysiology associated with this condition is yet to be fully understood.

Diffusion tensor imaging (DTI) is an advanced magnetic resonance imaging technique that measures *in vivo *tissue anisotropic diffusion properties [2-9]. DTI provides information not only about the magnitude, but also the directionality of water molecule displacement in tissue [5,10-12]. The effect of water molecules interacting with specific tissue structures is reflected in a diffusion image that can reveal the characteristics of the architectural organization in a physiological or pathological environment. In clinical research, DTI has been proven to be a sensitive and specific non-invasive imaging tool for determining brain damage and recovery in various neurological and pathological disorders [13-17]. Recently, DTI has also been applied in clinical studies of HCP to investigate the underlying mechanisms of injury associated with poor functional outcome [18-21]. However, the validation of DTI and the association of this non-invasive, macroscopic imaging biomarker with cellular level mechanisms for WM degeneration in HCP must be supported with cytopathology. This has yet to be established. To validate DTI as a biomarker for use in childhood HCP we have used a rat model [22] in which obstructive hydrocephalus can be induced reliably and cytopathology and neurobehavioral outcomes in normal, untreated, and treated hydrocephalic animals can be monitored closely and correlated at specific time points. The animal studies can be translated to the clinical setting by comparing common characteristic changes in DTI abnormalities in the context of the developing CNS. Ultimately, a better understanding of the tissue characteristics and mechanisms underlying DTI data will lead to more effective use of DTI in treatment decisions for the pediatric patient population.

The present study was designed to quantify the anisotropic diffusion properties in neonatal HCP rat brain using DTI and to investigate their association with underlying cytopathological changes. We hypothesized that the HCP rats will demonstrate abnormal anisotropic diffusion properties as measured by decreased fractional anisotropy (FA) and increased mean diffusivity (MD) when compared to the control group. We further hypothesized that these changes will correlate with underlying microstructural alterations seen in analysis of fixed tissue. The results of these studies will further our understanding of the use of DTI in the developing CNS and associated changes in HCP

## Methods

### Animals

Two groups of neonatal Sprague-Dawley rat pups were used in the study (Table 1). The first group included 12 rats with acquired hydrocephalus. The second group of controls consisted of 15 rats including 12 rats that received a sham injection of saline into the cisterna magna, plus 2 that were cooled in an ice-bath without injection, and 1 that was intact. None of the control rats developed hydrocephalus.

**Table 1:** Experimental procedures performed on each animal used in the study

	Days of Age											
	
Animal	P7	P8	P9	P10	P11	P12	P13	P15	P17	P19	P22	P23
**HCP**												

HCP_01		DTI								D†		

HCP_02			DTI									IHC*

HCP_03		DTI							D†			

HCP_04			DTI									IHC*

HCP_05					DTI							IHC

HCP_06				DTI				D†				

HCP_07					DTI/D							

HCP_08	DTI										IHC*	

HCP_09			DTI		IHC*							

HCP_10			DTI		IHC*							

HCP_11			DTI		IHC*							

HCP_12				DTI	IHC							

**Control**												

Control_01		DTI										IHC*

Control_02			DTI									IHC

Control_03		DTI										IHC

Control_04		DTI							D†			

Control_05	DTI										IHC	

Control_06			DTI								IHC	

Control_07			DTI								IHC*	

Control_08				DTI							IHC	

Control_09						DTI/D						

Control_10			DTI								IHC*	

Control_11					DTI							IHC

Control_12			DTI		IHC							

Control_13				DTI	IHC*							

Control_14				DTI	IHC							

Control_15			DTI		IHC*							

Note. DTI: diffusion tensor imaging; IHC: Sacrificed by cardiac perfusion and processed for immunohistochemistry; * underwent IHC; D: Died shortly after DTI scan; D†: Died shortly after scan, note the imaging data were not used due to severe HCP.

All animal procedures were performed according to the guidelines of the Institutional Animal Care and Use Committees at the Cincinnati Children's Hospital Research Foundation's and the University of Utah. The procedure to induce obstructive HCP has been described in detail in previous publications [22]. In brief, at P2, the animals were anesthetized by hypothermia and underwent injection of kaolin into the cisterna magna. MRI/DTI scans were performed between P7 and P12 at Cincinnati Children's Hospital Medical Center (CCHMC). At P11 (4 HCP rats, 4 control rats) or P22/23 (4 HCP rats, 9 control rats), the animals were euthanized and fixed by intracardiac perfusion. For this procedure, animals were first perfused with 0.9% normal saline followed by 4% (w/v) paraformaldehyde (PFA) in phosphate buffered saline through a left intraventricular injection. The brains were subsequently post-fixed in PFA and sent to the University of Utah for immunohistochemical evaluation and cytological analysis. Six rats (4/12 HCP rats, 2/15 controls) did not recover from anesthesia after the MRI/DTI scan. Although the respiration rate remained normal during the scan, these rats died under the anesthetic shortly after the scan and were not subjected to immunohistochemistry procedures (Table 1).

### Magnetic resonance and diffusion tensor imaging

Prior to scanning, the rats were anesthetized with 4% Isoflurane, placed in the supine position in the magnet, and maintained sedated with a mixture of 1-1.5% isoflurane and O_2 _for the duration of the experiment. They were kept warm, at approximately 37°C, with circulating warmed air. A bite bar was used to stabilize the head. The respiratory rate was monitored closely and maintained at 50 breaths/min (±10 breaths/min).

All MRI/DTI imaging data were acquired on a 7T Bruker MRI scanner (Bruker Biospec 70/30, Karlsruhe, Germany) equipped with an actively shielded 400 mT/m gradient set. The diameter of the gradient set was 20 cm. A custom-designed single-turn-solenoid (STS) RF coil was used in this study. The detailed feature of the coil has been previously reported [23]. The coil measured 25 mm in diameter and 30 mm in length. The B1 field distribution test showed that the inhomogeneity was modest with percentage deviation of the field strength in the peripheral region at <3% from the central value (Fig two in [23]).

DTI images were acquired in the coronal plane with a 6-direction diffusion weighted multi-slice spin-echo imaging protocol: TR = 2500 ms, TE = 21 ms, 5 slices, slice thickness = 1.5 mm, inter-slice gap = 0.25 mm, b-value = 1200 s/mm^2^, diffusion gradient duration δ = 4 ms, gradient separation Δ = 12 ms. Twenty-five of the 27 rats included in the present study were scanned at an in-plane resolution of 200 × 200 μm with four different fields of view (FOV) between 16 × 16 mm and 25.6 × 25.6 mm. No statistical significance was found between the hydrocephalic group and the control group in terms of the frequency of occurrence of these four different FOVs based on the Freeman-Halton extension of the Fisher exact probability test [24]. The time for acquisition ranged from 23:20 min to 37:20 min. The remaining two rats were scanned at slightly different in-plane resolutions (resolution = 196 × 196 μm, TA = 26:50 min., n = 1; resolution = 200 × 225 μm, TA = 23:20 min., n = 1) and no significant difference was identified with DTI measurement.

T2-weighted images of the whole brain were acquired using rapid acquisition with relaxation enhancement (RARE) sequence with the following parameters: TR = 1000 ms; effective TE = 70.56 ms; FOV = 32 × 19.2 × 19.2 mm; acquisition matrix = 256 × 128 × 128; spatial resolution = 0.125 × 0.150 × 0.150 mm; scanning time = 17:04 min. The T2-weighted RARE anatomical images were used to assess the ventricular size and also as a reference for anatomical structures.

### Immunohistochemistry

Brain tissue was processed from 2 control and 3 HCP rats sacrificed at P11 and 3 control and 3 HCP rats sacrificed at P22 or P23 (Table 1). Ten additional rats were perfused and fixed but did not undergo immunohistochemical analysis. Two control and 2 HCP rats sacrificed at P21 in previous experiments (not included in Table 1) were included for comparison in the present study. These additional animals underwent the same surgical procedures that have been described with the exception of the timing of kaolin injection or sham injection: the injection occurred at P1. They were not subjected to MRI/DTI scan thus were not included in the correlation analysis. Post-fixed brains were cut coronally into 3 blocks containing the frontal, parietal and occipital cortices. These blocks were embedded in paraffin, and sectioned serially at a thickness of 15 μm. Every 10^th ^section was selected in order to collect 12 sets of adjacent sections. Using established protocols, the following immunohistochemical stains were applied: (1) luxol fast blue (LFB), a sensitive staining method that detects mature myelin [25,26]; (2) glial fibrillary acidic protein (GFAP), a marker for both resting and reactive astrocytes [27]; and (3) ionized calcium-binding adaptor molecule (Iba-1), a marker for both resting and reactive microglia [28].

LFB staining was performed by de-paraffinizing and rehydrating the tissue. Slides were then incubated in the LFB stain for 2 h at 60°C. The sections were then rinsed in 95% ETOH, dH_2_O and differentiated with 0.05% lithium carbonate. Once the appropriate intensity of LFB stain was achieved, the sections were rinsed in distilled H_2_O, 70% ETOH, and then counterstained with cresyl violet.

Tissue sections were processed for GFAP immunohistochemistry according to previously described methods [29]. In summary, sections were rehydrated and antigen retrieval was performed. Prior to incubation (90 min) with the primary GFAP antibody (1:1000 Dako Cytomation, Glostrup, Denmark), sections were blocked with H_2_O_2_. The tissue was washed with phosphate buffered saline with 0.1% triton C (PBS-t) and the secondary antibody (1:200, Vector Laboratories, Burlingame, CA, USA) was applied for 45 min at room temperature. Following the secondary antibody Avidin Biotinylated enzyme Complex (ABC) was added for 30 min, washed with PBS-t and developed with diaminobenzidine (DAB). Sections were counterstained with cresyl violet.

Immunostaining for Iba-1 followed the same procedures described for GFAP with the exception that anti-Iba-1 (1: 500 WAKO Chemicals, Richmond, USA) was the primary antibody.

Stained sections were analyzed using a semi-quantitative scale described in detail in a recent report from our group [30]. Briefly, the scales pertaining to the GFAP and Iba-1 stains were based on cellular morphology and density. A score of 0 represented a resting state and a score of 3 represented a severely reactive state. The scale pertaining to the LFB stains was based on the intensity of the stained myelin. A score of 0 on the LFB scale represented a complete lack of myelin and a score of 3 indicated full myelination.

### DTI data processing and analysis

DTI data processing and analysis methodology details have been described in our previous publications [31,32]. The procedure is briefly summarized as follows. Image reconstructions, post-processing, and ROI-based DTI parameter calculations were performed with the software DTIStudio 2.4 [33]. On a pixel by pixel basis, the six elements (Dxx, Dyy, Dzz, Dxy, Dxz, and Dyz) were calculated and diagonalized to compute the three eigenvalues (λ_1_, λ_2_, λ_3_) corresponding to the three eigenvectors in the diffusion tensor matrix. Mean diffusivity (MD) was calculated as the mean of the three eigenvalues (MD = (λ_1 _+ λ_2 _+ λ_3_)/3). Fractional anisotropy (FA) was calculated on a pixel-wise basis, using standard methods [4,12].

**Figure 1:**
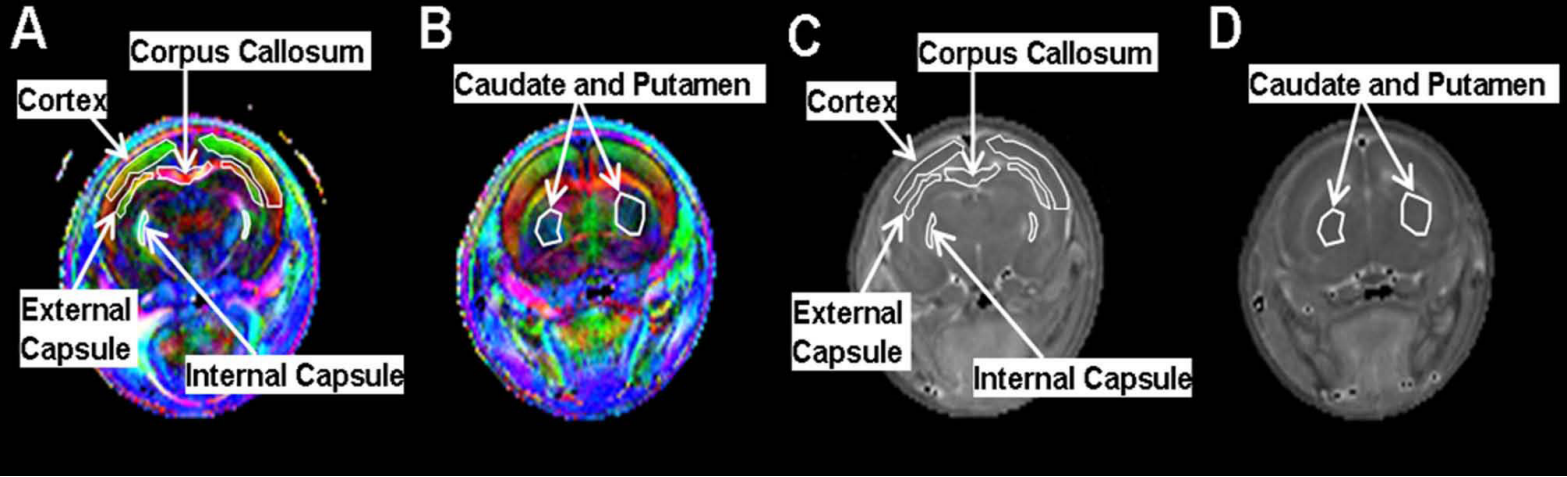
***In vivo *diffusion tensor imaging of a control rat at postnatal day 8**. In the fractional anisotropy-based color coded axis maps (A and B), the colors are used to indicate the preferred diffusion direction in a coronal plane. Red, green, and blue represent transverse, dorso-ventral and rostro-caudal directions, respectively. The brightness is coded by the FA value. On this map, both white matter (corpus callosum, internal capsule, and external capsule) and grey matter (cortex, caudate and putamen) can be identified. C and D are mean diffusivity maps showing the same structures.

**Figure 2:**
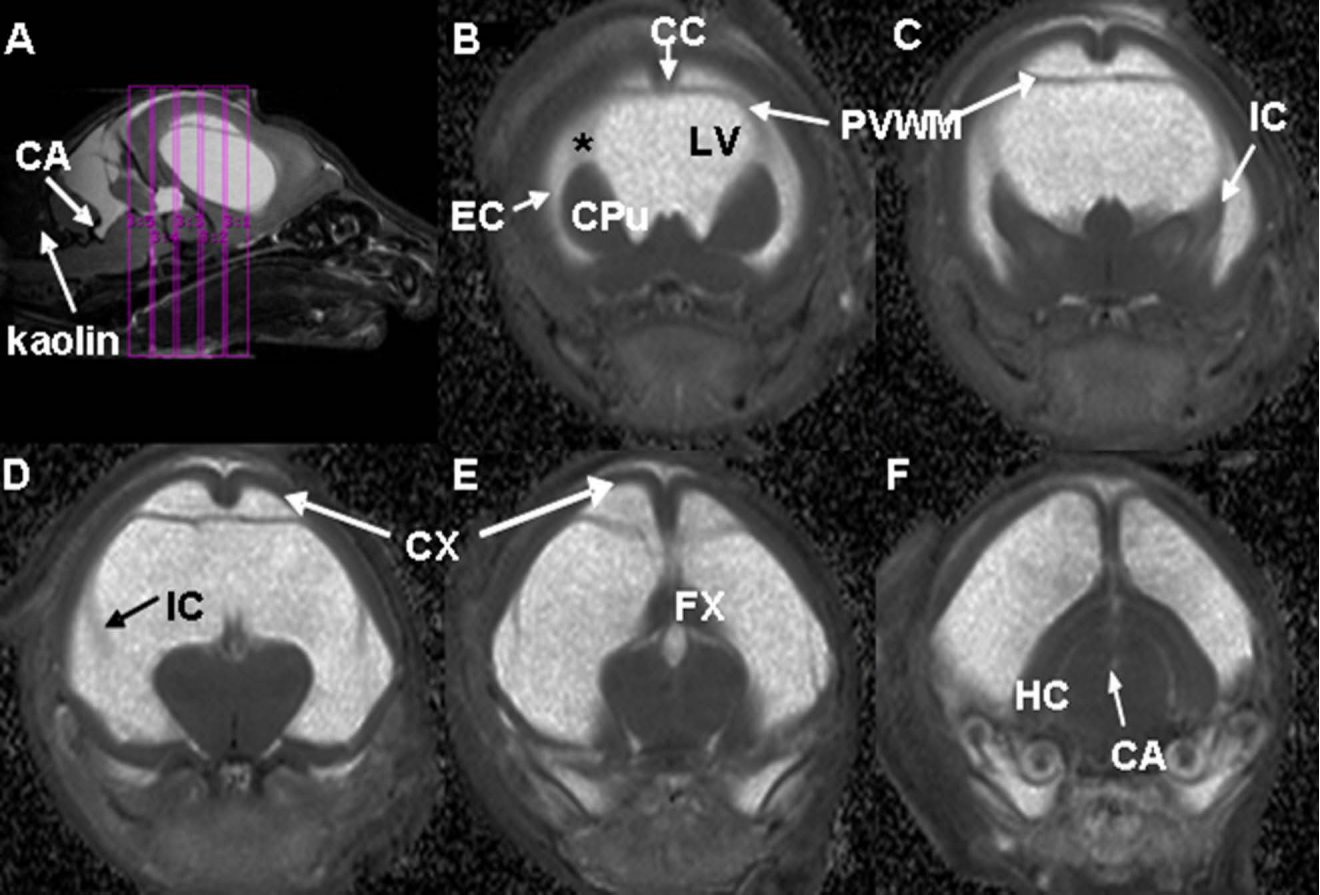
**Mean diffusivity maps of hydrocephalic rats at postnatal day 11**. The mid-sagittal image (A) shows the location of the five coronal slices (B-F) arranged from rostral to caudal. The expansion of the lateral ventricle and the posterior recess of the cerebral aqueduct (CA) can also be seen. Abbreviations: CC, corpus callosum; CPu, caudate-putamen; CX, cortex; EC, external capsule; FX, fornix; IC, internal capsule; HC, hippocampus; LV, lateral ventricle; PVWM, periventricular white matter.

As shown in Figure 1, a color-coded FA map was used to identify regions of interest (ROI). DTI parameters were calculated for respective ROIs individually in each section sampled. The ROIs for each subject were manually determined by an operator (WY) on color-coded FA and MD maps and anatomical images under the guidance of a neuroanatomist (JPM) and a pediatric neurosurgeon (FTM), (Figures 1 and 2). For each rat, the following ROIs were defined: corpus callosum (CC), internal capsule (IC), external capsule (EC), cerebral cortex (CX), caudate-putamen complex (CPu), hippocampus (HC), and fornix (FX). The ROI in the EC in hydrocephalic rats was found to be very difficult to delineate reliably, thus it was not included in the data analysis comparing HCP rats and control rats. EC of normal controls was still included in the data analysis when the age correlation of DTI parameters in various ROIs was studied in order to assess the developmental impact.

### Statistical analyses

Before conducting any statistical analysis, the distribution of the outcome data was examined for normality and the outcome variables that exhibit deviation from normality were transformed using a Box-Cox transformation [34]. For some of the outcome variables a suitable transformation was not found to satisfy the normality assumption. For uniformity we ran all statistical analysis using non-parametric methods. Wilcoxon two-sample test was used to compare the DTI parameters between HCP rats and the age matched controls over the period between P7 and P12. Although age is matched between the two groups, its potential impact on the outcome variables was examined using a Spearman rank correlation followed by regression analysis that contained both the comparison groups and age in the same model. We also ran a sub-group analysis using Wilcoxon two-sample test to compare the DTI parameters between HCP rats and controls at P9. Immunohistochemical results were also analyzed using Wilcoxon two-sample test. In addition, the Spearman correlation was used in the correlation analysis between DTI parameters and the immunochemistry ranking scales. To account for the expected proportion of incorrectly rejected null hypotheses (Type I error rate) due to multiple testing, we made a correction for multiple comparisons using the False Discovery Rate (FDR) method [35] in all the statistical tests except for the correlation analysis between DTI and cytopathology. All statistical analyses were done using SAS v.9.2 (SAS Institute Inc., Carey, NC, USA) or SPSS v.15 (Chicago, IL, USA).

## Results

### Abnormal DTI measurements in neonatal rats with hydrocephalus

**Figure 3:**
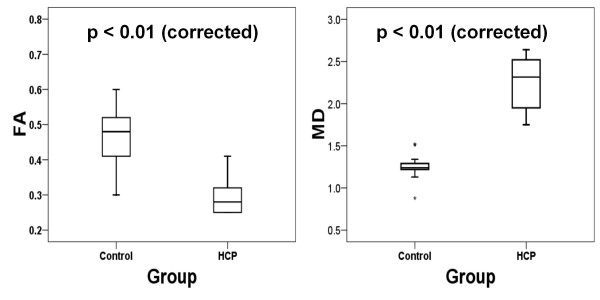
**Box-plots comparing the fractional anisotropy (FA) and mean diffusivity (MD, 10^-3^mm^2^/s) values in corpus callosum in hydrocephalic rats and the control rats**. All ages combined, n = 12 for hydrocephalic and n = 15 for control rats. The Wilcoxon two-sample test showed a significant difference (*p *< 0.05, corrected for multiple comparison).

DTI data acquired between P7 and P12 were compared between the control (n = 15) and the HCP group (n = 12). In the regression analysis, where comparison group and age were considered in the same model, age did not contribute significantly to the change in DTI measurements. Therefore, the two groups were compared with Wilcoxon independent two-sample test. Significant differences in DTI parameters were found in both GM and WM (Table 2, Figure 3). Among the GM ROIs, the FA of the HCP group in CX was significantly lower (*p *< 0.01) and the MD significantly higher (*p *< 0.05) than those in the control group. In the WM ROIs, statistically significant decreases in FA values (*p *< 0.01 for both CC and IC) and increases in MD values (*p *< 0.01 for both CC and IC) were found in the HCP group.

**Table 2:** Comparison of DTI parameters (fractional anisotropy, FA and mean diffusivity, MD) between hydrocephalic (HCP) and control groups between postnatal days 7 and 12

		HCP (n = 12)	Control (n = 15)	Corrected p-value
**CX**				
	**FA**	0.17 ± 0.03	0.25 ± 0.04	<0.01*
	**MD**	1.22 ± 0.09	1.12 ± 0.10	<0.05*
				
**CPu**				
	**FA**	0.22 ± 0.05	0.20 ± 0.05	NS
	**MD**	1.23 ± 0.10	1.17 ± 0.13	NS
				
**CC**				
	**FA**	0.29 ± 0.05	0.47 ± 0.08	<0.01*
	**MD**	2.25 ± 0.32	1.25 ± 0.15	<0.01*
				
**IC**				
	**FA**	0.23 ± 0.04	0.31 ± 0.04	<0.01*
	**MD**	2.39 ± 0.22	1.17 ± 0.09	<0.01*
				
**HC**				
	**FA**	0.19 ± 0.03	0.17 ± 0.04	NS
	**MD**	1.19 ± 0.10	1.10 ± 0.26	NS
				
**FX**				
	**FA**	0.20 ± 0.04	0.24 ± 0.05	NS
	**MD**	1.27 ± 0.10	1.18 ± 0.11	NS

Note: * significantly different (Wilcoxon two-sample test). Numbers are mean ± standard deviation. MD is in the unit of 10^-3^mm^2^/s. NS: not significant, brain regions as for Figure 2.

To explore the earliest time at which the impact of HCP can be reflected in DTI parameters, we compared FA and MD values between the two groups for each individual postnatal day. Only the difference at P9 reached statistical significance (Table 3). The Wilcoxon two-sample tests were performed for all DTI values in each ROI as described in the Methods section. The FA values of the CX in the HCP group were significantly lower compared to the controls (*p *< 0.05). In the WM, specifically the CC, the FA in HCP rats was significantly lower than that in the controls (*p *< 0.05) and the MD was significantly increased (*p *< 0.05). In the IC, the MD value was abnormally high in the HCP rats (*p *< 0.05), but their FA values were within normal range. In the FX, the FA values of the HCP rats were abnormally high (*p *< 0.05) with the MD value within normal range. No statistically significant difference was identified for the other two ROIs (CPu, HC, Table 3).

**Table 3:** Comparison of DTI parameters (fractional anisotropy, FA and mean diffusivity, MD) between HCP and control groups at postnatal day 9

		HCP (n = 5)	Control (n = 6)	Corrected p-value
**CX**				
	**FA**	0.16 ± 0.01	0.25 ± 0.05	<0.05*
	**MD**	1.22 ± 0.07	1.17 ± 0.14	NS
				
**CPu**				
	**FA**	0.22 ± 0.07	0.21 ± 0.06	NS
	**MD**	1.26 ± 0.10	1.21 ± 0.17	NS
				
**CC**				
	**FA**	0.28 ± 0.03	0.44 ± 0.07	<0.05*
	**MD**	2.44 ± 0.18	1.27 ± 0.13	<0.05*
				
**IC**				
	**FA**	0.24 ± 0.03	0.28 ± 0.05	NS
	**MD**	2.37 ± 0.17	1.21 ± 0.15	<0.05*
				
**HC**				
	**FA**	0.2 ± 0.04	0.17 ± 0.04	NS
	**MD**	1.25 ± 0.04	0.94 ± 0.45	NS
				
**FX**				
	**FA**	0.20 ± 0.05	0.28 ± 0.03	<0.05*
	**MD**	1.26 ± 0.05	1.21 ± 0.10	NS

Note: * significantly different (Wilcoxon two sample test). Numbers are mean ± standard deviation. MD, is in the unit of 10^-3^mm^2^/s. NS: not significant, brain regions as for figure 2.

### Immunohistochemical results

**Figure 4:**
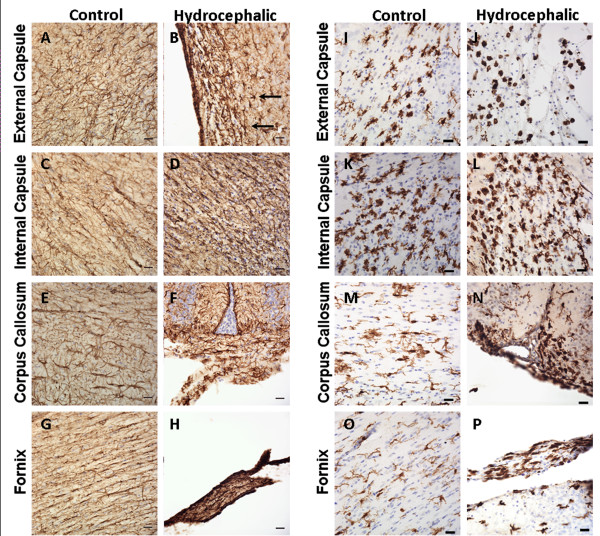
**Representative photomicrographs of GFAP for astrocytes (A-H) and Iba-1 for microglia (I-P) immunohistochemistry from the external capsule, internal capsule, corpus callosum, and fornix in P11 saline control animals (A, C, E, G for GFAP; I, K, M, O for Iba-1) and hydrocephalic animals (B, D, F, H for GFAP; J, L, N, P for Iba-1)**. For GFAP, despite the immaturity of the tissue, the hydrocephalic animals exhibit the same severe glial reaction observed in the P22/23 animals as evidenced by large intensely stained cell bodies and thick processes (B, straight arrows). For Iba-1, note the round intensely stained profiles that represent highly phagocytic microglial somata in the white matter of control animals. These profiles are increased in hydrocephalic animals at this age, but are not found in any 22 or 23-day old animals. Scale bar = 25 μm and cresyl violet counterstain for all panels.

**Figure 5:**
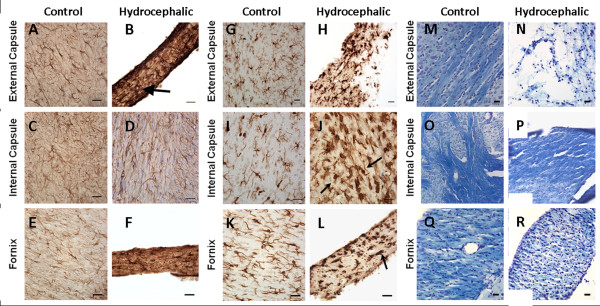
**Representative photomicrographs of GFAP (A-F), Iba-1 (G-L), and LFB (M-R) immunohistochemistry of the external capsule, internal capsule, and the fornix of P22/23 control (A, C, & E for GFAP; G, I, and K for Iba-1; M, O, & Q for LFB) and hydrocephalic animals (B, D, & F for GFAP; H, J, & L for Iba-1; N, P, & R for LFB)**. GFAP staining in the hydrocephalic animals reveals severe reactive astrocytosis when compared to the saline control animals. Panel A shows typical characteristics of resting astrocytes that includes lightly stained cell bodies with thin processes. Panel B demonstrates distinctive characteristics of reactive astrocytes including a darkly stained cell body with thick processes (arrow). For Iba-1, control animals (G, I, & K) exhibit resting microglial cells which are characterized by a small unremarkable cell body with thin processes. The hydrocephalic animals (H, J, & L) display reactive microglia cells which are typified by a darkly stained, enlarged, distorted cell body with thick processes (arrows, J and L). At the end stages of reactivity they exhibit a "balled up" appearance known to represent a phagocytic state (arrow, L). LFB staining is dark and uniform in the saline controls which indicates the preservation of myelin (M, O, & Q). Whereas, the hydrocephalic animals exhibit a more diffuse and variable staining pattern, providing evidence of demyelination most likely resulting from the mechanical stress that accompanies HCP (N, P, & R). Scale bar = 25 μm for all panels except O and P, scale bar = 50 μm.

Overall, hydrocephalic animals at both P11 (Figure 4) and P22/23 (Figure 5) displayed increases in astrocyte and microglial responses and a decrease in myelination in all WM regions studied. Numerous GFAP- and Iba-1-positive cells were found, and many of these astrocytes and microglia exhibited the large soma and thickened processes characteristic of reactive glia.

Quantitative rankings (Table 4) confirmed a trend of increased astrocyte reactivity (GFAP staining) at P11 in IC, EC, FX, and CC. At P22/P23 the same regions reached statistical significance (*p *< 0.05), with the exception of the corpus callosum which could not be analyzed as it was too thin or missing altogether.

**Table 4:** Cytological rankings in hydrocephalic and control rats at P11 and P22/P23 as determined from immunohistochemical analysis.

		P11			P22/P23		
		**HCP (n = 3)**	**Control (n = 2)**	**Corrected p-value**	**HCP (n = 5)**	**Control (n = 5)**	**Corrected *p*-value**

**IC**							
	**GFAP**	2.67 ± 0.58	0.5 ± 0.00	NS	2.80 ± 0.28	0.25 ± 0.30	<0.05*
	**Iba-1**	2.67 ± 0.29	2.0 ± 0.00	NS	2.90 ± 0.22	0.90 ± 0.58	<0.05*
	**LFB**	n/d	n/d	n/d	1.69 ± 0.51	2.80 ± 0.21	<0.05*
							
**EC**							
	**GFAP**	3.00 ± 0.00	0.69 ± 0.27	NS	3.00 ± 0.00	0.18 ± 0.14	<0.05*
	**Iba-1**	3.00 ± 0.00	1.5 ± 0.00	NS	3.00 ± 0.00	0.40 ± 0.39	<0.05*
	**LFB**	n/d	n/d	n/d	1.60 ± 1.04	2.15 ± 0.73	NS
							
**FX**							
	**GFAP**	3.00 ± 0.00	0.88 ± 0.53	NS	2.75 ± 0.35	0.20 ± 0.33	<0.05*
	**Iba-1**	3.00 ± 0.00	0.5 ± 0.00	NS	2.85 ± 0.34	0.15 ± 0.14	<0.05*
	**LFB**	n/d	n/d	n/d	1.35 ± 0.60	2.20 ± 0.45	NS
							
**CC**							
	**GFAP**	3.00 ± 0.00	0.75 ± 0.35	NS	n/d	n/d	n/d
	**Iba-1**	3.00 ± 0.00	0.75 ± 0.35	NS	n/d	n/d	n/d
	**LFB**	n/d	n/d	n/d	n/d	n/d	n/d

GFAP: glial fibrillary acidic protein for astrocytes, Iba-1: ionized calcium-binding adaptor molecule for microglia, LFB: luxol fast blue for myelin. Note. * significantly different (Wilcoxon two sample test); NS: not significant; n/d: not detectable; LFB stain too weak at P11 and CC too thin or missing at P22/P23. Brain regions as for figure 2.

Microglial reactivity followed the same trend of increases in the IC, EC, FX, and CC (Table 4). At P22/P23, microglial reactivity also reached statistical significance (*p *< 0.05) with the exception of the CC which could not be analyzed.

Myelination, as visualized with LFB staining, was undetectable in both the control and hydrocephalic animals at P11. At P22/P23 myelination had matured to the point where it could be detected histologically, and although there was a consistent trend toward less staining in the IC, EC, and FX of hydrocephalic animals these changes were only statistically significant in the IC, *p *< 0.05 (Figure 5 and Table 4).

### Correlation between immunohistochemical rankings and DTI

The immunohistochemical semi-quantitative scale [30] was used to correlate cytology to imaging findings (Table 5). In the IC and FX, no significant differences were identified between the DTI measurement and any of the 3 ranked immunohistochemical parameters at P11. The FA in the CC was negatively correlated with the P11 GFAP ranking (R = -0.89; *p *< 0.05) and moderately correlated with the P11 Iba-1 ranking but without statistical significance (R = -0.78). Also at P11, the MD in the CC was moderately correlated with GFAP but without statistical significance (R = 0.78) and was significantly correlated with Iba-1 ranking scale (R = 0.89, *p *< 0.05, Table 5).

**Table 5:** Correlation analysis between DTI metrics and immunohistochemical ranking scores.

		IC				Fornix				CC			
		
		P11		P22/P23		P11		P22/P23		P11		P22/P23	
		
		R	*p*	R	*p*	R	*p*	R	*p*	R	*p*	R	*p*
**GFAP**													
	**FA**	-0.47	NS	-0.88	<0.05*	-0.48	NS	-0.52	NS	-0.89	<0.05*	n/d	n/d
	**MD**	0.63	NS	0.79	NS	-0.18	NS	0.52	NS	0.78	NS	n/d	n/d
													
**Iba-1**													
	**FA**	-0.26	NS	-0.88	<0.05*	-0.45	NS	-0.62	NS	-0.78	NS	n/d	n/d
	**MD**	0.79	NS	0.76	NS	-0.24	NS	0.49	NS	0.89	<0.05*	n/d	n/d
													
**LFB**													
	**FA**	n/d	n/d	0.74	NS	n/d	n/d	-0.03	NS	n/d	n/d	n/d	n/d
	**MD**	n/d	n/d	-0.74	NS	n/d	n/d	-0.40	NS	n/d	n/d	n/d	n/d

GFAP: glial fibrillary acidic protein for astrocytes, Iba-1: ionized calcium-binding adaptor molecule for microglia, LFB: luxol fast blue for myelin. Note: * significant correlation between DTI and immunohistochemical grading (Spearman test); 3 HCP and 2 control rats were used at P11; 3 HCP and 3 control rats were used at P22/P23; n/d: not detectable.

The ventricular enlargement in the third week and thereafter was too severe to allow for reliable imaging, hence the histology results acquired at P22/P23 were correlated to the DTI metrics measured between P7-P10. The FA values in the IC were found to be significantly correlated with GFAP and Iba-1 (*p *< 0.05, for both) suggesting perhaps that radiographic evidence of injury in this anatomic location may occur early in the process. The correlation between FA in the IC and LFB staining of the same area was moderate but it did not reach statistical significance. MD values measured from the IC were found to be moderately correlated with the rankings of all three stains but without reaching statistical significance (Table 5).

## Discussion

The goal of the present study was twofold: (1). to show proof-of-principle for adopting DTI methods to study the impact of obstructive HCP on the brain in a rodent model; and (2) to evaluate the tissue integrity in the hydrocephalic brain using DTI and correlate it with cytopathology. Our results demonstrate that DTI can successfully be used to quantify the underlying structural abnormality as reflected in the changes of the regional anisotropic diffusion properties in both GM and WM; even on a sub-millimeter scale in rat brain. In addition, these results show that the DTI findings have a positive correlation to the histopathological changes in HCP rats. Thus we establish that DTI parameters may be used as a surrogate marker to evaluate cerebral tissue integrity during the development of HCP, further supporting its use in translational research studies in the clinical setting.

As summarized by Beaulieu *et al*. [6], anisotropic diffusion properties are strongly influenced by the micro-structural components within the brain. The deviation of diffusion indices based on DTI from the normal range is believed to be an indication of the integrity, or the lack thereof, in various structural components, such as the myelin sheath or axonal membrane. Neuronal degeneration is often reported to be a reflection of decreased FA accompanied by an increased MD [36,37]. Conversely, increased FA accompanied by decreased radial diffusivity and increased axial diffusivity is often regarded as an indication of compression of tissue, as seen in space-occupying lesions, such as non-invasive tumors [38]. In clinical DTI studies of pediatric hydrocephalus, different patterns of change in DTI indices have been described in specific WM regions [18-21]. For example, infants with HCP have been reported to demonstrate abnormally low FA in the corpus callosum and abnormally high FA in the internal capsule [20]. Similarly, older patients (>7 yrs) also showed analogous patterns of diffusion [18]. We speculate that multiple injury mechanisms may coexist in the HCP patient. The clinical manifestation of injury in HCP may depend on various factors such as the location of the structure under study, and the intrinsic tissue resistance, as well as the timing and duration of HCP during critical time points of CNS development. Among the four WM regions of interest examined in the present study (Tables 2 and 3), the EC in most HCP rats was unidentifiable using DTI maps. This can be considered an indication of severe tissue damage. Even though anatomically identifiable during the preparation for immunohistochemical staining, partial volume effect prevented us from making meaningful measurements in this region in any of the HCP rats. In the CC and the IC, significant decreases in FA and increases in MD values were found in rats with HCP. The FX showed similar DTI trends even though the difference did not reach statistical significance. These group differences based on data averaged over time (between P7 and P12) can be found as early as P9 when the comparison was made for individual post natal days. This may serve as preliminary evidence of the early impact of HCP as reflected from the DTI measurement. As described earlier, a decrease in FA usually indicates degenerative damage in the myelin or axonal membrane [6], a change that is regarded as less reversible than a mere physical compression which often presents as an increase in FA [38,39]. Combining the early timing and the potential nature of the changes, our results support early management in the treatment of HCP. In the clinical setting it would translate to having a pre-operative MRI demonstrating a significant reduction in FA values from our standard ROIs [20,21].

DTI is seldom used to quantify the anisotropy in GM structures. However, relatively high anisotropy has been reported previously in the neonatal brain of both experimental models and clinical studies [39-41]. In the normal individual, the elevated FA is followed by a rapid decrease until a plateau is reached in adulthood. This change in anisotropy is attributed to the developmental change in neuronal proliferation/migration, apoptosis and axonal pruning, and synaptogenesis occurring during the postnatal period [39]. In the present study, DTI was used to determine the anisotropic diffusion properties in three GM structures: the CX, CPu, and HC. When comparing our data to the FA values at a corresponding age reported by Bockhorst *et al *[39], our FA measurements in normal rats were similar in the CX but higher than previously reported in the HC and CPu. In the present study, HCP rats had a significantly reduced FA (from 0.25 to 0.17) in the CX, indicating that the impact of HCP not only affects the adjacent periventricular WM and the subcortical WM, but also the GM structures that are further peripherally located within the cranial vault.

It should be stressed that the DTI measurement is an objective but indirect reflection of the underlying micro-structural integrity or pathological condition under study. As a non-invasive imaging biomarker, DTI results need to be examined and correlated to the histopathological data which is regarded as the gold standard for studying the injury mechanisms at the cellular level. In this study, we examined GFAP immunoreactivity to characterize the temporal-spatial changes of reactive gliosis following HCP. Our results from GFAP stains showed that a strong glial reaction was seen in the HCP rats in the IC, EC, and FX at both P11 and P22/P23. Astrocytosis was also present at P11 in the CC. The correlation analysis of DTI with our GFAP ranking showed a moderate to strong correlation in the CC at P11. Likewise, the microglial reaction (Iba-1) also demonstrated a moderate to strong correlation with the DTI measurements in the CC at P11. LFB staining was very weak at P11 in control animals; this result is expected given that these observations occurred only three days after the initiation of myelin maturation (P7) [39,42,43]. Consequently, the paucity of LFB staining in hydrocephalic brains at P11 was difficult to interpret. At P22/P23, even though the reduction of myelination was, as expected, statistically significant in IC in HCP rats (Table 4), only a moderate correlation (without statistical significance) was found between the LFB staining in IC at P22/P23 and the DTI measurement at P7-P9 (Table 5).

In the present study, astrocytes and microglial cells demonstrated an early and continuous response to CNS injury in untreated HCP. The correlations between DTI and those cellular changes reveal initial evidence for the potential of DTI to serve as an imaging biomarker in studying the progression of HCP.

The results presented in this study add new important value to the field of advanced imaging in experimental HCP. However, caution should be exercised in the interpretation of the results due to the overall small sample size. Our data do not allow for a more comprehensive analysis to evaluate the causal effect or the predicting power of DTI. Moreover, it is possible that partial volume effects may have artificially increased the differences in diffusivity measurements between the groups. Within a 1.5 mm thick slice as used in the study, the structures examined may have changed diffusion direction. The impact of this potential confounding factor will be reduced by increasing the imaging resolution using 3 D fast DTI imaging sequence, e.g., EPI or RARE based DTI sequence. With regard to the animal model, the current study will benefit if the progression of ventricular enlargement can be controlled and managed both in time and in the degree of severity. We have begun experiments to address these shortcomings. In addition, monitoring the longitudinal changes between the pre- and post-shunting conditions will further increase our understanding of the underlying injury mechanisms as well as the course for recovery.

## Conclusion

The current study demonstrates that DTI is a sensitive tool in the investigation of brain structural integrity of the neonatal rat with hydrocephalus. Significant changes of diffusion properties (increased diffusivity and decreased anisotropy) occurred in the HCP rats in both GM and WM. DTI alterations significantly correlate with changes in astrocytosis, microgliosis, and myelination. These findings indicate that DTI is a useful imaging biomarker for non-invasive investigation of tissue injury and recovery in hydrocephalus.

## List of abbreviations

CC: corpus callosum; CPu: caudate-putamen complex; CX: cortex; DTI: diffusion tensor imaging; EC:external capsule; FA: fractional anisotropy; FX: fornix; GFAP: glial fibrillary acidic protein; GM: grey matter; HC: hippocampus; HCP: hydrocephalus; Iba-1: ionized calcium binding adaptor molecule; IC: internal capsule; LFB: luxol fast blue; MD: mean diffusivity; WM: white matter.

## Competing interests

The authors declare that they have no competing interests.

## Authors' contributions

WY: conception and design, data processing and analysis, interpretation of data, manuscript drafting, revision, and finalizing; KED: data acquisition, data processing and analysis, manuscript revision; JPM: conception and design, data analysis, interpretation of data, manuscript drafting, revision, and finalizing; SKH: conception and design, interpretation of data, manuscript drafting and revision; DML: conception and design, data acquisition, manuscript drafting and revision; AC: data acquisition, manuscript drafting and revision; MM: data acquisition, data analysis, manuscript revision; AS: data acquisition, data processing, manuscript revision; DH: data acquisition, data interpretation, manuscript drafting and revision; MA: data analysis, manuscript revision; FTM: conception and design, data analysis, interpretation of data, manuscript drafting, revision, and finalizing.

All authors have read and approved the final version of the manuscript.

## References

[B1] Hirsch JF Surgery of hydrocephalus: past, present and future Acta Neurochir (Wien) 1992 116 155 160 10.1007/BF01540869 1502950

[B2] Douek P Turner R Pekar J Patronas N Le Bihan D MR color mapping of myelin fiber orientation J Comput Assist Tomogr 1991 15 923 929 10.1097/00004728-199111000-00003 1939769

[B3] Basser PJ Mattiello J LeBihan D Estimation of the effective self-diffusion tensor from the NMR spin echo J Magn Reson B 1994 103 247 254 10.1006/jmrb.1994.1037 8019776

[B4] Basser PJ Mattiello J LeBihan D MR diffusion tensor spectroscopy and imaging Biophys J 1994 66 259 267 10.1016/S0006-3495(94)80775-1 8130344 PMC1275686

[B5] Beaulieu C Allen PS Determinants of anisotropic water diffusion in nerves Magn Reson Med 1994 31 394 400 10.1002/mrm.1910310408 8208115

[B6] Beaulieu C The basis of anisotropic water diffusion in the nervous system - a technical review NMR Biomed 2002 15 435 455 10.1002/nbm.782 12489094

[B7] Horsfield MA Jones DK Applications of diffusion-weighted and diffusion tensor MRI to white matter diseases - a review NMR Biomed 2002 15 570 577 10.1002/nbm.787 12489103

[B8] Neil J Miller J Mukherjee P Huppi PS Diffusion tensor imaging of normal and injured developing human brain - a technical review NMR Biomed 2002 15 543 552 10.1002/nbm.784 12489100

[B9] Sotak CH The role of diffusion tensor imaging in the evaluation of ischemic brain injury - a review NMR Biomed 2002 15 561 569 10.1002/nbm.786 12489102

[B10] Moseley ME Cohen Y Kucharczyk J Mintorovitch J Asgari HS Wendland MF Tsuruda J Norman D Diffusion-weighted MR imaging of anisotropic water diffusion in cat central nervous system Radiology 1990 176 439 445 2367658 10.1148/radiology.176.2.2367658

[B11] Henkelman RM Stanisz GJ Kim JK Bronskill MJ Anisotropy of NMR properties of tissues Magn Reson Med 1994 32 592 601 10.1002/mrm.1910320508 7808260

[B12] Pierpaoli C Jezzard P Basser PJ Barnett A Di Chiro G Diffusion tensor MR imaging of the human brain Radiology 1996 201 637 648 8939209 10.1148/radiology.201.3.8939209

[B13] Rosenberger G Kubicki M Nestor PG Connor E Bushell GB Markant D Niznikiewicz M Westin CF Kikinis R A JS Age-related deficits in fronto-temporal connections in schizophrenia: a diffusion tensor imaging study Schizophr Res 2008 102 181 188 10.1016/j.schres.2008.04.019 18504117 PMC2684860

[B14] Werring DJ Clark CA Barker GJ Thompson AJ Miller DH Diffusion tensor imaging of lesions and normal-appearing white matter in multiple sclerosis Neurology 1999 52 1626 1632 10331689 10.1212/wnl.52.8.1626

[B15] Werring DJ Toosy AT Clark CA Parker GJ Barker GJ Miller DH Thompson AJ Diffusion tensor imaging can detect and quantify corticospinal tract degeneration after stroke J Neurol Neurosurg Psychiatry 2000 69 269 272 10.1136/jnnp.69.2.269 10896709 PMC1737065

[B16] Huisman TA Schwamm LH Schaefer PW Koroshetz WJ Shetty-Alva N Ozsunar Y Wu O Sorensen AG Diffusion tensor imaging as potential biomarker of white matter injury in diffuse axonal injury AJNR Am J Neuroradiol 2004 25 370 376 15037457 PMC8158566

[B17] Wilde EA Chu Z Bigler ED Hunter JV Fearing MA Hanten G Newsome MR Scheibel RS Li X Levin HS Diffusion tensor imaging in the corpus callosum in children after moderate to severe traumatic brain injury J Neurotrauma 2006 23 1412 1426 10.1089/neu.2006.23.1412 17020479

[B18] Assaf Y Ben-Sira L Constantini S Chang LC Beni-Adani L Diffusion tensor imaging in hydrocephalus: initial experience AJNR Am J Neuroradiol 2006 27 1717 1724 16971621 PMC8139798

[B19] Hasan KM Eluvathingal TJ Kramer LA Ewing-Cobbs L Dennis M Fletcher JM White matter microstructural abnormalities in children with spina bifida myelomeningocele and hydrocephalus: A diffusion tensor tractography study of the association pathways J Mag Res Imag 2008 27 700 709 10.1002/jmri.21297 PMC3046030 18302204

[B20] Yuan W Mangano FT Air EL Holland SK Jones BV Altaye M Bierbrauer K Anisotropic diffusion properties in infants with hydrocephalus: a diffusion tensor imaging study AJNR Am J Neuroradiol 2009 30 1792 1798 10.3174/ajnr.A1663 19661167 PMC7051526

[B21] Air EL Yuan W Holland SK Jones BV Bierbrauer K Altaye M Mangano FT Longitudinal comparison of pre- and postoperative diffusion tensor imaging parameters in young children with hydrocephalus J Neurosurg Pediatr 2010 5 385 391 10.3171/2009.11.PEDS09343 20367345

[B22] McAllister JP Maugans TA Shah MV Truex RC Jr Neuronal effects of experimentally induced hydrocephalus in newborn rats J Neurosurg 1985 63 776 783 10.3171/jns.1985.63.5.0776 4056881

[B23] DiFrancesco MW Rasmussen JM Yuan W Pratt R Dunn S Dardzinski BJ Holland SK Comparison of SNR and CNR for in vivo mouse brain imaging at 3 and 7 T using well matched scanner configurations Med Phys 2008 35 3972 3978 10.1118/1.2968092 18841848 PMC2809704

[B24] Freeman GH Halton JH Note on an exact treatment of contingency, goodness of fit and other problems of significance Biometrika 1951 38 141 149 14848119

[B25] Del Bigio MR Wilson MJ Enno T Chronic hydrocephalus in rats and humans: white matter loss and behavior changes Ann Neurol 2003 53 337 346 10.1002/ana.10453 12601701

[B26] Khan OH Enno TL Del Bigio MR Brain damage in neonatal rats following kaolin induction of hydrocephalus Exp Neurol 2006 200 311 320 10.1016/j.expneurol.2006.02.113 16624304

[B27] Miller JM McAllister JP Reduction of astrogliosis and microgliosis by cerebrospinal fluid shunting in experimental hydrocephalus Cerebrospinal Fluid Res 2007 4 5 10.1186/1743-8454-4-5 17555588 PMC1899521

[B28] Ito D Imai Y Ohsawa K Nakajima K Fukuuchi Y Kohsaka S Microglia-specific localisation of a novel calcium binding protein, Iba1 Brain Res Mol Brain Res 1998 57 1 9 10.1016/S0169-328X(98)00040-0 9630473

[B29] Deren KE Forsyth J Abdullah O Hsu EW Klinge PM Silverberg GD Johanson CE McAllister JP Low levels of amyloid-beta and its transporters in neonatal rats with and without hydrocephalus Cerebrospinal Fluid Res 2009 6 4 10.1186/1743-8454-6-4 19470163 PMC2689851

[B30] Deren KE Packer M Forsyth J Milash B Abdullah OM Hsu EW McAllister JP Reactive astrocytosis, microgliosis and inflammation in rats with neonatal hydrocephalus Exp Neurol 2010 226 110 119 10.1016/j.expneurol.2010.08.010 20713048

[B31] Yuan W Holland SK Schmithorst VJ Walz NC Cecil KM Jones BV Karunanayaka P Michaud L Wade SL Diffusion tensor MR imaging reveals persistent white matter alteration after traumatic brain injury experienced during early childhood AJNR Am J Neuroradiol 2007 28 1919 1925 10.3174/ajnr.A0698 17905895 PMC4295209

[B32] Yuan W Holland SK Jones BV Crone K Mangano FT Characterization of abnormal diffusion properties of supratentorial brain tumors: a preliminary diffusion tensor imaging study J Neurosurg Pediatr 2008 1 263 269 10.3171/PED/2008/1/4/263 18377300

[B33] Jiang H van Zijl PC Kim J Pearlson GD Mori S DtiStudio: resource program for diffusion tensor computation and fiber bundle tracking Comput Methods Programs Biomed 2006 81 106 116 10.1016/j.cmpb.2005.08.004 16413083

[B34] Box GEP Cox DR An analysis of transformations J R Stat Soc, Ser B 1964 26 211 252

[B35] Benjamini Y Hochberg Y Controlling the false discovery rate: a practical and powerful approach to multiple testing J Roy Stat Soc Ser B 1995 57 1919 1925

[B36] Wang J Wai Y Lin WY Ng S Wang CH Hsieh R Hsieh C Chen RS Lu CS Microstructural changes in patients with progressive supranuclear palsy: a diffusion tensor imaging study J Magn Reson Imaging 2010 32 69 75 10.1002/jmri.22229 20578012

[B37] Boska MD Hasan KM Kibuule D Banerjee R McIntyre E Nelson JA Hahn T Gendelman HE Mosley RL Quantitative diffusion tensor imaging detects dopaminergic neuronal degeneration in a murine model of Parkinson's disease Neurobiol Dis 2007 26 590 596 10.1016/j.nbd.2007.02.010 17428671 PMC2040046

[B38] Schonberg T Pianka P Hendler T Pasternak O Assaf Y Characterization of displaced white matter by brain tumors using combined DTI and fMRI Neuroimage 2006 30 1100 1111 10.1016/j.neuroimage.2005.11.015 16427322

[B39] Bockhorst KH Narayana PA Liu R Ahobila-Vijjula P Ramu J Kamel M Wosik J Bockhorst T Hahn K Hasan KM Perez-Polo JR Early postnatal development of rat brain: in vivo diffusion tensor imaging J Neurosci Res 2008 86 1520 1528 10.1002/jnr.21607 18189320

[B40] McKinstry RC Mathur A Miller JH Ozcan A Snyder AZ Schefft GL Almli CR Shiran SI Conturo TE Neil JJ Radial organization of developing preterm human cerebral cortex revealed by non-invasive water diffusion anisotropy MRI Cereb Cortex 2002 12 1237 1243 10.1093/cercor/12.12.1237 12427675

[B41] Maas LC Mukherjee P Carballido-Gamio J Veeraraghavan S Miller SP Partridge SC Henry RG Barkovich AJ Vigneron DB Early laminar organization of the human cerebrum demonstrated with diffusion tensor imaging in extremely premature infants Neuroimage 2004 22 1134 1140 10.1016/j.neuroimage.2004.02.035 15219585

[B42] Hamano K Iwasaki N Takeya T Takita H A quantitative analysis of rat central nervous system myelination using the immunohistochemical method for MBP Brain Res Dev Brain Res 1996 93 18 22 10.1016/0165-3806(96)00025-9 8804688

[B43] Hamano K Takeya T Iwasaki N Nakayama J Ohto T Okada Y A quantitative study of the progress of myelination in the rat central nervous system, using the immunohistochemical method for proteolipid protein Brain Res Dev Brain Res 1998 108 287 293 10.1016/S0165-3806(98)00063-7 9693804

